# Arthritis imaging using a near-infrared fluorescence folate-targeted probe

**DOI:** 10.1186/ar1483

**Published:** 2005-01-14

**Authors:** Wei-Tsung Chen, Umar Mahmood, Ralph Weissleder, Ching-Hsuan Tung

**Affiliations:** 1 Center of Molecular Imaging Research, Massachusetts General Hospital, Harvard Medical School, Charlestown, Massachusetts, USA; 2 Radiology Department, Taipei Municipal Jen-Ai Hospital, Taipei, Taiwan

**Keywords:** arthritis, fluorescence, folate receptor, folic acid, near-infrared, optical imaging

## Abstract

A recently developed near-infrared fluorescence-labeled folate probe (NIR2-folate) was tested for *in vivo *imaging of arthritis using a lipopolysaccharide intra-articular injection model and a KRN transgenic mice serum induction mouse model. In the lipopolysaccharide injection model, the fluorescence signal intensity of NIR2-folate (*n *= 12) and of free NIR2 (*n *= 5) was compared between lipopolysaccharide-treated and control joints. The fluorescence signal intensity of the NIR2-folate probe at the inflammatory joints was found to be significantly higher than the control normal joints (up to 2.3-fold, *P *< 0.001). The NIR2-free dye injection group showed a persistent lower enhancement ratio than the NIR2-folate probe injection group. Excessive folic acid was also given to demonstrate a competitive effect with the NIR2-folate. In the KRN serum transfer model (*n *= 4), NIR2-folate was applied at different time points after serum transfer, and the inflamed joints could be detected as early as 30 hours after arthritogenic antibody transfer (1.8-fold increase in signal intensity). Fluorescence microscopy, histology, and immunohistochemistry validated the optical imaging results. We conclude that *in vivo *arthritis detection was feasible using a folate-targeted near-infrared fluorescence probe. This receptor-targeted imaging method may facilitate improved arthritis diagnosis and early assessment of the disease progress by providing an *in vivo *characterization of active macrophage status in inflammatory joint diseases.

## Introduction

Rheumatoid arthritis (RA) is a common chronic inflammatory and destructive arthropathy that consumes substantial personal, social, and economic costs. The synovial membrane in patients with RA is characterized by hyperplasia, by increased vascularity, and by an infiltration of inflammatory cells, including activated macrophages [1]. Activated macrophages presenting in large numbers of arthritic joints play an active role in RA [2] and other inflammatory diseases [3] by producing cytokines that drive subsequent inflammatory reaction.

Folate receptor (FR) is a 38-kDa glycosyl phosphatidylinositol-anchored protein that binds the vitamin folic acid with high affinity (< 1 nM) [4,5]. With the exception of the kidney and the placenta, normal tissues express low or undetectable levels of FR [4]. Previously it has been reported that FR has three isoforms: FR-α, FR-β, and FR-γ. Among them, FR-β, a nonepithelial isoform of FR, is expressed on activated synovial macrophages but not on resting synovial macrophages [6]. Folate derivatization might therefore be exploited to target activated macrophages involved in inflammatory joint disease. Turk and colleagues [7,8] have recently used folate-^99m^Tc for assaying the participation of activated macrophages in an adjuvant-induced arthritis model, and have shown that folate-^99m^Tc selectively targets activated macrophages. This suggests that folate-linked imaging agents warrant further scrutiny as possible tools for evaluating arthritis.

A newly synthesized folic acid and near-infrared fluorochrome conjugate (NIR2-folate) was recently used as a FR-targeting imaging probe *in vivo *[9,10]. Fluorescence in the near-infrared spectrum (700–900 nm) was used for *in vivo *imaging because it allows efficient photon migration through the tissues and has minimal autofluorescence [11]. The use of near-infrared fluorescent (NIRF) *in vivo *imaging probes has been shown to significantly enhance tumor detection [12-15], to facilitate identification of small preneoplastic lesions [16], and to allow objective assessment of new therapeutic paradigms [17] in animal studies. The NIRF imaging technology has recently been extended to arthritic studies. *In vivo *NIRF imaging of arthritis in experimental animals was demonstrated using a protease-sensitive probe and NIRF-labeled antibody [18-21]. The goal of the present study is to determine whether a fairly abundant FR on activated macrophages in the arthritic inflammatory process could serve as a target for NIRF-enhanced optical imaging.

## Materials and methods

### Imaging probe

The folate-targeting optical probe NIR2-folate, consisting of a near-infrared fluorochrome (NIR2) and folic acid, was synthesized and characterized as previously described [9,10]. Briefly, folic acid was first reacted with 2,2'-(ethylenedioxy) bis(ethylamine) using di-isopropylcarbodimide as the coupling agent in dimethyl sulfoxide. The *N*-hydroxysuccinimide-activated ester of NIR2 [22] was then coupled with the amino-derivatized folic acid. The final conjugate was purified by C-18 reverse-phase HPLC and confirmed by mass spectroscopic analysis. The NIR2-folate has an excitation wavelength maximum at 662 nm and an emission wavelength maximum at 686 nm.

### Animal preparation and arthritis models

All animal studies were approved by the Institutional Animal Care Committee. Carbon dioxide inhalation was used for euthanasia. C57BL/6 mice (Jackson Laboratory, Bar Harbor, ME, USA) weighing 19–21 g, 12 weeks old, were handled in accordance with government guidelines. Lipopolysaccharide (LPS) intra-articular injection and KRN transgenic mice serum transfer served as two mice arthritis models in this study.

The LPS induction arthritis model was achieved according to published protocols [23,24]. Mice were anesthetized with ketamine (90 mg/kg) and xylazine (10 mg/kg) intraperitoneally, and then LPS (Sigma, St Louis, MO, USA), 10 μg in 20 μl saline, was injected intra-articularly into the right ankle joint through the Achilles tendon using a 30-gauge needle. As a control, the same volume of normal saline was injected in the opposite ankle joint of the same animal.

The KRN transgenic mice were a gift from Dr D Mathis and Dr C Benoist (Joslin Diabetes Center, Boston, MA, USA). Blood was obtained from arthritic adult KRN mice, and the sera containing arthritogenic autoantibodies were pooled [18,25,26]. One hundred micoliters of KRN mice serum were intravenously injected into healthy C57BL/6 mice, and the NIR2-folate probe was then given at different time points after serum transfer to detect early inflammatory changes.

### Experimental groups

In the LPS induction model, the three experimental groups of animals were injected intravenously with NIR2-folate probe (2 nmol per animal, *n *= 12), with free NIR2 (2 nmol per animal, *n *= 5), or with 600-fold of folic acid (1200 nmol per animal) 5 min prior to NIR2-folate probe injection (2 nmol per animal, *n *= 5) to demonstrate the competition effect of free folic acid against the probe. In the KRN serum transfer model, four animals were intravenously injected with 100 μl KRN serum and the NIR2-folate probe was given 24 hours (*n *= 1) or 96 hours (*n *= 3) after serum transfer.

### *In vivo *NIRF reflectance imaging and lesion assessment

All animals were imaged in a prone position using a home-built NIRF reflectance imaging system, which has been described elsewhere [27]. For fluorescence acquisition, a 615–645 nm excitation filter and a 680–720 nm emission filter (Omega Optical, Brattleboro, VT, USA) were used. Images were analyzed using commercially available software (Digital Science 1D software; Kodak, Rochester, NY, USA). Following data acquisition, postprocessing and visualization were performed using the in-house program CMIR Image. The enhancement ratio of the inflamed joint was used to demonstrate the effectiveness of the probe, which was defined by the fluorescence signal intensity (SI) at the affected ankle joint divided by the fluorescence SI at the opposite ankle joint. NIRF images were acquired preinjection and postinjection at different time points.

### Histology, immunohistochemistry, and immunofluorescent microscopy assessment

Ankles were excised and fixed in phosphate-buffered formalin for 24 hours, and were subsequently decalcified in 10% EDTA for 48 hours, paraffin embedded, cut into 8-μm sections, and stained with H&E. Immunohistochemistry was performed using an anti-activated macrophage antibody [28] (Mac-3, 1:500 dilution, rat anti-mouse monoclonal antibody; BD Biosciences, San Diego, CA, USA) and a goat anti-human folate receptor polyclonal antibody (sc-16387, 1:100 dilution; Santa Cruz Biotechnology, Santa Cruz, CA, USA), revealed with biotinylated rabbit anti-rat and donkey anti-goat secondary antibodies (1:250 dilution; Santa Cruz Biotechnology). The staining procedure was performed with a modified avidin–biotin–peroxidase complex technique. The slides were visualized with a chromogen of diaminobenzidine (Vectastain; Vector Laboratories, Burlingame, CA, USA). Sections were counterstained with hematoxylin (Vector Laboratories). Positive immunoreactions appeared as dark brown staining on a blue background. Control sections were processed identically but with incubation of the nonspecific isotype immunoglobulin (Vector Laboratories).

Immunofluorescence staining was performed using Mac-3 rat anti-mouse monoclonal antibody (1:500 dilution) and FITC-conjugated anti-rat secondary antibody (1:250 dilution; Vector Laboratories). The inflamed ankles were cut into 10-μm thick slices using a Leica CM 1900 cryotome (Leica, Bannockburn, IL, USA). Slices were analyzed using an inverted epifluorescence microscope (Axiovert; Zeiss, Thorn-Wood, NY, USA). FITC and Cy5.5 channels were used for Mac-3 and NIR2-folate fluorescence signal detection. A cooled CCD camera (Sensys; Photometrics, Tucson, AZ, USA) adapted with a bandpass filter was used for image capture, and IPLab software (Scanlytics, Fairfax, VA, USA) was used for image analysis.

### Statistical analysis

Data are presented as the mean and standard error of the mean. Statistical analysis of the fluorescence SI and the enhancement ratio between different groups was conducted using a two-tailed paired Student *t *test. The paired Student *t *test was used for analyzing the SI difference between bilateral ankles in the same mouse. *P *< 0.05 was considered to indicate a statistically significant difference. All statistics were analyzed using Stata 7.0 (Stata, College Station, TX, USA) for Windows (Microsoft, Redmond, WA, USA).

## Results

### Establishment of a LPS-induced arthritis model

**Figure 1:**
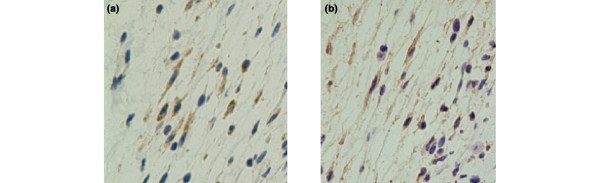
Immunoperoxidase staining of **(a) **Mac-3 and **(b) **folate receptor (FR) at an arthritic ankle 72 hours after lipopolysaccharide induction. The Mac-3-positive and FR-positive cells morphologically correlated well in adjacent tissue sections. Magnification, 400 ×.

Progressive discoloration and swelling of the ankle joints was noted 24 hours after LPS intra-articular injection. Abundant polymorphonuclear cell infiltration was noted in the synovial lining layer and the subsynovial adipose tissue in histologic sections 48 hours after LPS injection. Immunohistochemistry revealed Mac-3-positive and FR-positive cells scattered among polymorphonuclear cells and subsynovial tissues in adjacent tissue sections (Fig. 1). These findings indicate that arthritis can be induced by LPS, and that the presence of active macrophages within inflammatory tissues can be used as a target for the NIR2-folate probe.

### NIRF imaging of a LPS-induced mice arthritis model

**Figure 2:**
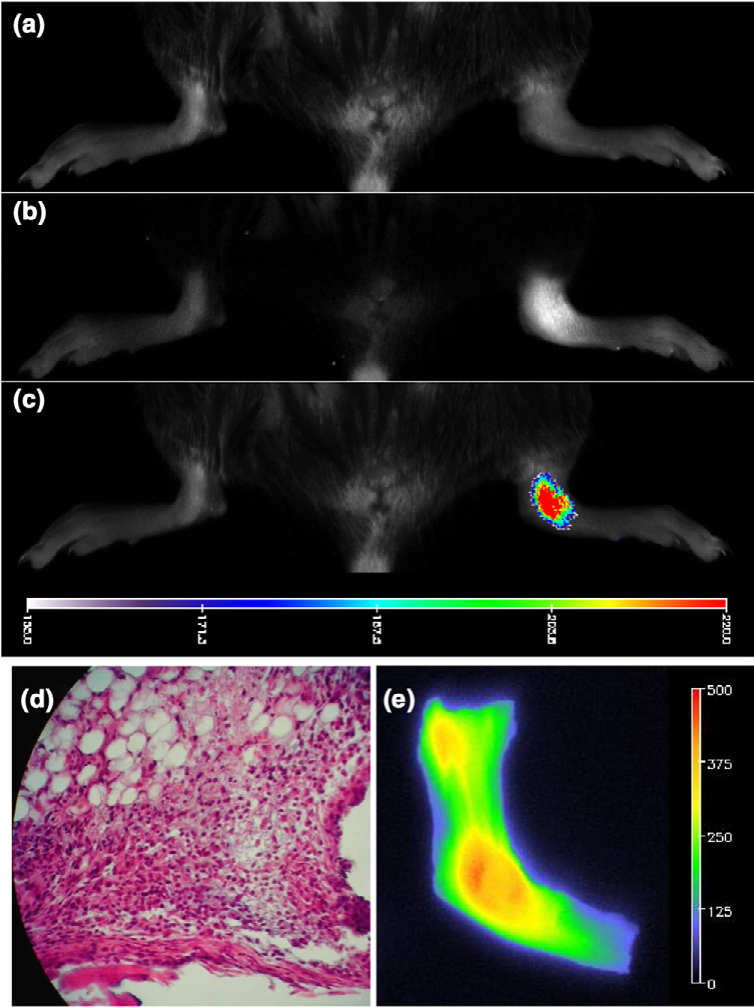
*In vivo *near-infrared fluorescent (NIRF) imaging of inflammatory joints in the lipopolysaccharide (LPS) induction model. The NIR2-folate probe was intravenously injected 2 days after LPS intra-articular injection. **(a) **White-light images obtained 48 hours after intra-articular LPS injection at the right ankle joint; soft tissue swelling was noted at the affected joint. **(b) **NIRF images obtained 24 hours after NIR2-folate injection. Note the strong fluorescence signal in the LPS-treated ankle compared with the opposite control side (enhancement ratio = 2.31). **(c) **A merged NIRF signal with a white-light image showing specific increased fluorescence signal intensity at the affected joint. **(d) **H&E-stain section of the right ankle joint showing abundant inflammatory cell infiltration at subsynovial tissues. Original magnification, 100 ×. **(e) **NIRF images of a longitudinal section of the LPS-treated ankles. Pseudo-color coding was used to demonstrate the stronger fluorescence signal surrounding the ankle joint.

The NIR2-folate probe was injected 48 hours after LPS induction (*n *= 12). The fluorescence SI of the inflamed joints was significantly higher than the opposite ankle joint at 2 min, and 12, 24, 48, and 72 hours after probe injection (468 ± 51 arbitrary units [AU] versus 303 ± 33 AU, 400 ± 31 AU versus 181 ± 18 AU, 310 ± 18 AU versus 137 ± 8 AU, 209 ± 14 AU versus 111 ± 7 AU, and 144 ± 14 AU versus 80 ± 4 AU; *P *< 0.001 in all sets) (Fig. 2). There was no significant difference in the preinjection fluorescence SI in bilateral ankle joints (85 ± 6 AU versus 82 ± 7 AU, *P *> 0.05).

**Figure 3:**
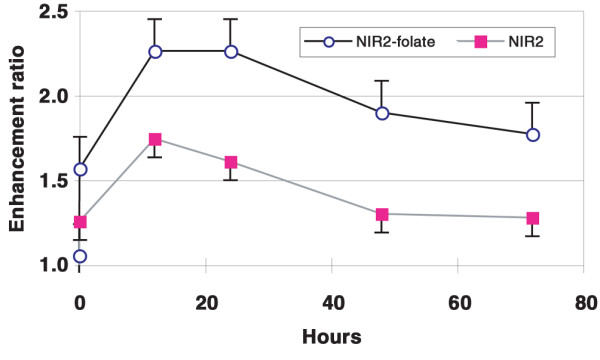
Enhancement ratio of lipopolysaccharide (LPS)-treated inflamed ankles in NIR2-folate (*n *= 12) and NIR2-free dye (*n *= 5) injection groups at different time points. A significantly higher enhancement ratio was noted in the NIR2-folate injection group at 24-hour, 48-hour and 72-hour time points (*P *< 0.05).

The average enhancement ratio of the inflamed joint was up to 2.3-fold in the first 12 and 24 hours after probe injection, and remained at 1.8-fold 72 hours after probe injection (Fig. 3). In comparison, the NIR2-free dye group (*n *= 5) showed a persistent lower enhancement ratio than the probe injection group at all time points (Fig. 3). The average enhancement ratios of the inflamed ankles in the NIR2-free dye group and the NIR2-folate group at 24-hour, 48-hour, and 72-hour time points were 1.6 ± 0.1 versus 2.3 ± 0.1, 1.3 ± 0.1 versus 1.9 ± 0.1, and 1.3 ± 0.03 versus 1.8 ± 0.1 (*P *< 0.05), respectively. To understand the possible mechanism, folic acid was used to compete with the probe. In the folic acid competition study (*n *= 5), 600-fold folic acid (1.2 μm per animal) was given intravenously 5 min before the NIR2-folate injection. The enhancement ratio of the arthritic joint in the folic acid competition group was significantly lower than that of the NIR2-folate injection group (1.1 ± 0.1 versus 1.6 ± 0.1, *P *< 0.05).

### Colocalization of NIRF signal with Mac-3 immunofluorescence

**Figure 4:**
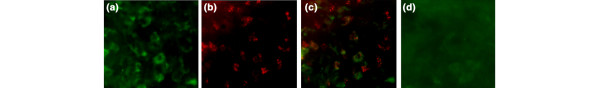
Colocalization of Mac-3-positive cells and NIR2-folate uptake cells in a lipopolysaccharide-induced arthritic ankle 48 hours after NIR2-folate injection. **(a) **Immunofluorescence staining for activated macrophage revealed in the FITC channel. **(b) **NIR2-folate uptake cells are revealed in the near-infrared fluorescent channel. **(c) **Superimposed image shows Mac-3-positive cells colocalized well with NIR2-folate cellular origins. **(d) **A negative control without primary antibody. Original magnification, 400 ×.

Immunofluorescence of the LPS-treated arthritic joint showed scattered Mac-3-positive cells in the inflammatory tissues in the FITC channel (Fig. 4a), whereas NIR2-folate uptake cells were seen in the near-infrared channel using an inverted epifluorescence microscope (Fig. 4b). In the superimposed image (Fig. 4c), the Mac-3-positive cells colocalized well with NIR2-folate uptake cells.

### Establishment of a KRN serum transfer mice arthritis model

**Figure 5:**
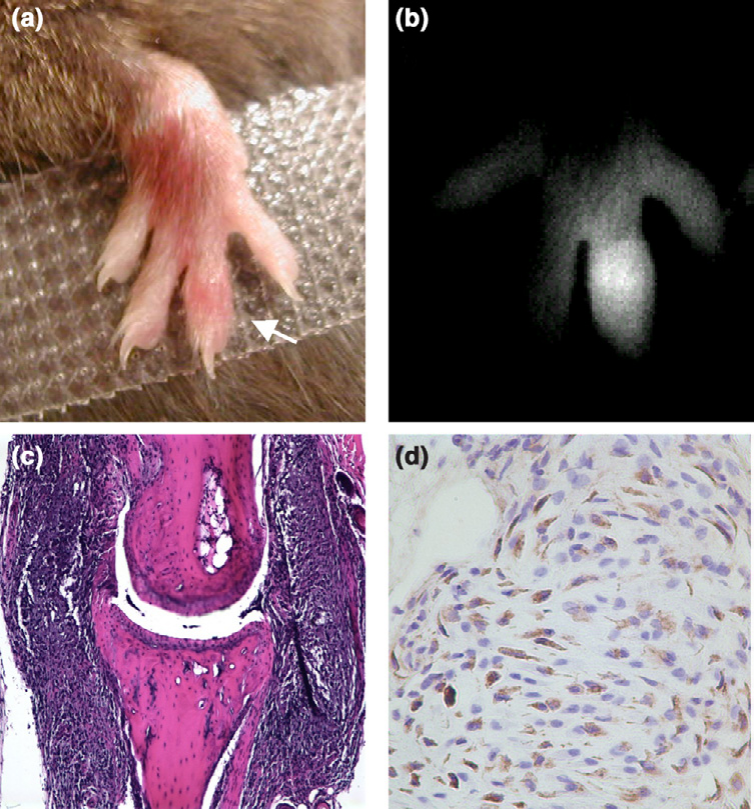
Establishment of the KRN serum transfer model. **(a) **Discoloration and swelling (arrow) of the right third proximal interphalangeal joint is noted in a healthy C57BL/6 mouse 4 days after KRN serum transfer. **(b) **Near-infrared fluorescent imaging of the right paw showed increase fluorescence signal intensity at the inflammatory joint (enhancement ratio = 1.9). **(c) **Correlated H&E-stain section showed abundant inflammatory cells infiltration with pannus-like formation. Original magnification, 100 ×. **(d) **Immunoperoxidase staining of Mac-3. Mac-3-positive cell infiltration among polymorphonuclear cells was noted in the pannus. Original magnification, 400 ×.

There was no visible swelling or discoloration at peripheral joints in the first 2 days after KRN serum transfer. Progressive discoloration and swelling of the peripheral joints was noted 3 days after serum transfer in sick KRN mice (Fig. 5a). In histological sections, Mac-3-positive cells intermingled among polymorphonuclear cells, and pannus formation was noted in the affected joints (Fig. 5b,c).

### NIRF imaging of a KRN serum transfer mice arthritic model

NIR2-folate was first given intravenously 4 days after KRN mice serum transfer. At this time point, discoloration and swelling of the affected peripheral joints was clearly observed (Fig. 5a). An intense fluorescence signal was found in peripheral joints (Fig. 5d). The NIR signal of the affected joints was 1.5-fold to 3.5-fold (average, 2.4-fold) higher than that of the unaffected joints.

**Figure 6:**
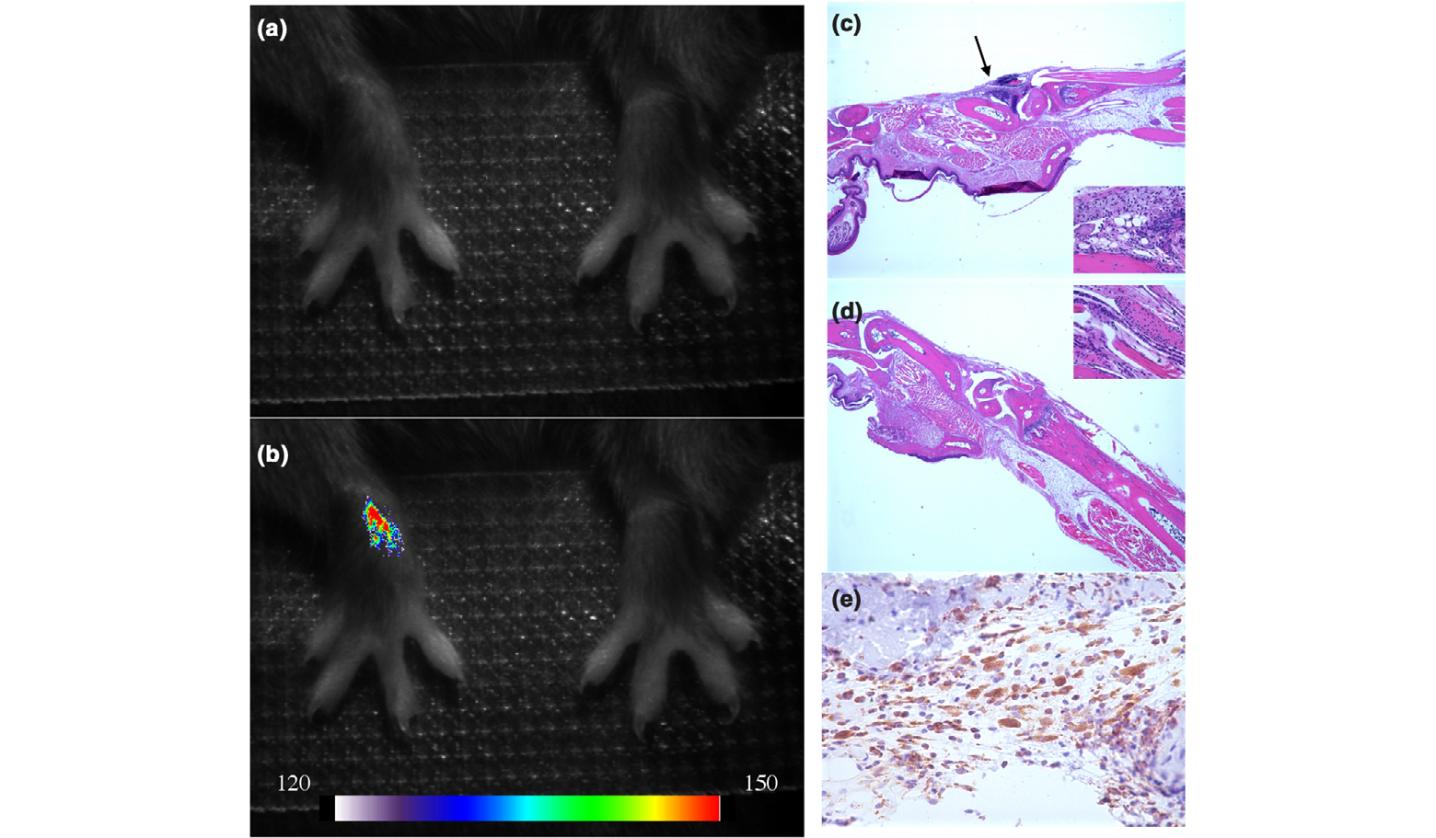
Early detection (30 hours after KRN serum transfer) of the inflammatory joint by NIR2-folate. **(a) **White-light image showed no remarkable swelling at bilateral paws. **(b) **Merged near-infrared fluorescent signal with a white-light image showed increase fluorescence signal intensity at the dorsal aspect of the right wrist, which has a 1.8-fold increase compared with the left wrist. **(c) **H&E-stain histology of the right wrist showed polymorphonuclear cell infiltration at the dorsal aspect of the right wrist (arrow). Magnification, 20 × (400 ×, insert). **(d) **Histology of the left wrist showed no remarkable inflammatory cell infiltration. Magnification, 20 × (400 ×, insert). **(e) **Immunohistochemistry of the right wrist showed Mac-3-positive cell infiltration at subsynovial tissues. Magnification, 400 ×.

To evaluate its ability for early detection of the inflammatory process, NIR2-folate was then given intravenously at a much earlier time point – 24 hours after serum transfer. No gross swelling or discoloration at peripheral joints could be observed (Fig. 6a). Six hours after the NIR2-folate probe injection (30 hours after serum transfer), however, the NIRF reflectance imaging showed a 1.8-fold increase in the fluorescence signal at the right wrist joint as compared with the opposite site (Fig. 6b). The correlated histology showed an increased amount of inflammatory cells at the affected joint compared with the opposite wrist (Fig. 6c,d). Abundant Mac-3-positive cell infiltration at the right wrist joint region was also revealed by immunohistochemistry (Fig. 6e).

## Discussion

Activated macrophages are thought to be intimately involved in the pathogenesis of RA by directly destroying articular tissue, secreting metalloproteinases, and attracting or activating other immune cells via the release of cytokines [2,29]. The quantitation of activated macrophages in joint tissues might consequently be of diagnostic value because activated macrophage content correlates well with articular destruction and poor disease prognosis in humans [2,30]. Because FR expression may coincide with macrophage activation [6], we hypothesized that arthritic joints could be imaged using folate-derivatized fluorescent imaging agents. The present studies demonstrated that the folate-targeted NIRF probe can indeed selectively target activated macrophages *in vivo*, and that folate-linked imaging agents can facilitate the noninvasive analysis of inflammatory activity *in situ*.

Two different animal arthritis models were used in this study. The LPS induction model was established by intra-articular injection of LPS, which induces transient synoviocyte hyperplasia and polymorphonuclear cell infiltration [23,24,31,32]. The advantage of the LPS induction model is that the opposite ankle joint could be used as an internal control, thus demonstrating the effectiveness of the probe in statistical analysis. The entity of this model, however, is a bacterial toxin-induced arthritis that resembles pyogenic arthritis instead of RA. The second model was established by transferring serum of sick KRN mice into healthy B6 mice, which induces synovial polymorphonuclear cells and macrophage infiltration by arthritogenic immunoglobulins [18,26,33]. The KRN serum transferred model resembles human RA because both are chronic symmetric joint diseases with pannus formation and destructive bone and cartilage erosion, predominantly of the distal joints.

The enhancement ratio of inflamed joints in the LPS model was slightly increased in the NIR2-free dye injection group during the first 24 hours after NIR2 injection. This might be due to nonspecific phagocytosis by activated macrophages, or due to NIR2-free dyes pooled at the interstitial space because of increased vascular permeability at the inflammation tissues. However, the enhancement ratio of the inflammatory joints in the NIR2-folate injection group was significantly higher than that of NIR2 injection group, which was more prominent 48 hours after injection (Fig. 3). Most of the NIR2-free dye began to be washed out from the inflamed joints, but NIR2-folate remained at the inflamed joints 72 hours after injection. The data indicate that the NIR2-folate probe has significant advantages over nonspecific fluorochromes for *in vivo *imaging, the latter often being used for nontargeted image enhancement [34,35].

Histological colocalization of the infiltrated Mac-3-positive and FR-positive cells was found to correlate well in the inflammatory tissues (Fig. 1). The NIR2-folate uptake cells colocalized with Mac-3-positive cells using fluorescence microscopy (Fig. 4), which indicates that uptake of folate conjugates at inflammatory joints is mediated by activated macrophages. In addition, the *in vivo *competition study confirmed that free folate was able to compete with the NIR2-folate probe for FR binding. The average enhancement ratio of arthritic joints in the folic acid competition group was significantly lower than in the NIR2-folate group postadministration. The results support the fluorescent probe uptake being receptor dependent.

Another important finding of this study is the potential of applying this technique in early assessment of RA. Our results indicate that the folate-linked NIR fluorescence probe could detect mild inflammatory changes as early as 30 hours after arthritogenic antibody transfer, before any morphological changes can be observed. A sensitive imaging modality for assessment of early events in RA could provide valuable information for diagnosis and treatment [36].

^99m^Tc-folate has recently been used to assay the participation of activated macrophages in adjuvant-induced arthritis mice models using gamma scintigraphy as the imaging modality [7]. In contrast, optical imaging is a noninvasive method and does not depend on radiolabeled contrast agents such as those in nuclear medicine; there is thus no exposure of the patient to ionizing radiation. The present hindrance of optical imaging is that tissue penetration of light in living tissue may attenuate the SI. The near-infrared fluorescence probe allows the most efficient photon migration through the tissues [11]. In addition, there is less soft tissue around peripheral joints, which gives the near-infrared optical imaging a competitive role in the diagnosis of peripheral joint disease, especially in detection of early arthritis or assessment of treatment effects.

## Conclusions

The results indicate that it is feasible to image the activated macrophage status in inflamed joints *in vivo *at an early stage. The FR-targeting probe not only offers better assessment at early stages in inflammatory disease, but also improves the evaluation of future anti-inflammatory treatments. This technique may therefore represent a step toward the level of molecular diagnosis of arthritis.

## Abbreviations

AU = arbitrary units; FITC = fluorescein isothiocyanate; FR = folate receptor; H&E = hematoxylin and eosin; HPLC = high-performance liquid chromatography; LPS = lipopolysaccharide; NIRF = near-infrared fluorescent; RA = rheumatoid arthritis; SI = signal intensity.

## Competing interests

The author(s) declare that there are no competing interests.

## Authors' contributions

WC and CT participated in all experimental design, data collection and analysis, and drafted the manuscript. UM participated in the KRN experiments and drafted the manuscript. RW participated in the design and helped to draft the manuscript. All authors read and approved the final manuscript.
